# Automated Implant Placement Pathway from Dental Panoramic Radiographs Using Deep Learning for Preliminary Clinical Assistance

**DOI:** 10.3390/diagnostics15202598

**Published:** 2025-10-15

**Authors:** Pei-Yi Wu, Shih-Lun Chen, Yi-Cheng Mao, Yuan-Jin Lin, Pin-Yu Lu, Kai-Hsun Yu, Kuo-Chen Li, Tsun-Kuang Chi, Tsung-Yi Chen, Patricia Angela R. Abu

**Affiliations:** 1 Department of Periodontics, Division of Dentistry, Taoyuan Chang Gung Memorial Hospital, Taoyuan City 33305, Taiwan; q384@cgmh.org.tw; 2 Department of Electronic Engineering, Chung Yuan Christian University, Taoyuan City 320317, Taiwan; 3 Department of Operative Dentistry, Taoyuan Chang Gung Memorial Hospital, Taoyuan City 33305, Taiwan; louiszzzzz@cgmh.org.tw; 4 Program on Semiconductor Manufacturing Technology, Academy of Innovative Semiconductor and Sustainable Manufacturing, National Cheng Kung University, Tainan City 701401, Taiwan; m28121562@gs.ncku.edu.tw; 5 Department of Information Management, Chung Yuan Christian University, Taoyuan City 320317, Taiwan; 11244230@o365st.cycu.edu.tw (P.-Y.L.); 11244226@o365st.cycu.edu.tw (K.-H.Y.); kuochen@cycu.edu.tw (K.-C.L.); 6 Department of Electrical Engineering, Ming Chi University of Technology, 84 Gungjuan Rd., New Taipei City 243303, Taiwan; 7 Department of Electronic Engineering, Feng Chia University, Taichung City 40724, Taiwan; tsungychen@fcu.edu.tw; 8 Ateneo Laboratory for Intelligent Visual Environments, Department of Information Systems and Computer Science, Ateneo de Manila University, Quezon City 1108, Philippines; pabu@ateneo.edu

**Keywords:** AI-assisted diagnostic, image enhancement, implant placement pathway, You only Look Once

## Abstract

**Background/Objective:** Dental implant therapy requires clinicians to identify edentulous regions and adjacent teeth accurately to ensure precise and efficient implant placement. However, this process is time-consuming and subject to operator bias. To address this challenge, this study proposes an AI-assisted detection framework that integrates deep learning and image processing techniques to predict implant placement pathways on dental panoramic radiographs, supporting clinical decision-making. **Methods:** The proposed framework is first applied to YOLO models to detect edentulous regions and employs image enhancement techniques to improve image quality. Subsequently, YOLO-OBB is utilized to extract pixel-level positional information about neighboring healthy teeth. An implant pathway orientation visualization algorithm is applied to derive clinically relevant implant placement recommendations. **Results:** Experimental evaluation using YOLOv9m and YOLOv8n-OBB demonstrated stable performance in both recognition and accuracy. The models achieved Precision values of 88.86% and 89.82%, respectively, with an average angular error of only 1.537° compared to clinical implant pathways annotated by dentists. **Conclusions:** This study presents the first AI-assisted diagnostic framework for DPR-based implant pathway prediction. The results indicate strong consistency with clinical planning, confirming its potential to enhance diagnostic accuracy and provide reliable decision support in implant dentistry.

## 1. Introduction

Dental implants are widely regarded as a predictable and long-term solution for tooth replacement [1,2]. However, the success of treatment strongly depends on accurate implant placement pathway selection during preoperative planning [3]. Misaligned implant pathways may lead to severe complications, including cortical bone fenestration, maxillary sinus perforation, mandibular nerve injury, and increased risk of implant failure [4]. Several clinical studies have emphasized that many of these adverse outcomes are associated with inadequate diagnosis or insufficient planning, underscoring the need for precise radiographic evaluation of the implant site [5,6]. Conventional imaging modalities such as periapical radiographs (PA) [7], dental panoramic radiographs (DPRs) [8], and cone-beam computed tomography (CBCT) [9] provide essential diagnostic information, but each has limitations in terms of dimensional accuracy, radiation exposure, or interpretability. To overcome these challenges, recent research has highlighted the potential of artificial intelligence (AI) to automate the interpretation of dental radiographs and assist in planning implant pathways with higher precision and reproducibility. In clinical practice, commercially available solutions such as coDiagnostiX [10] and implant systems like BEGO Implants [11], supported through the services of BEGO Medical’s Scanning and Design Center, exemplify how digital tools can integrate with AI-driven approaches to enhance diagnostic accuracy and surgical planning.

The success and longevity of dental implants are influenced by a wide range of internal and external factors, including the patient’s systemic health, lifestyle habits, local oral conditions and prosthetic design. Due to the complexity of these variables [12], treatment planning based solely on clinicians’ experience carries potential subjectivity and error. This undermines the predictability of outcomes and highlights the importance of adopting more systematic, automated, and standardized approaches [13]. Studies have reported deviations of up to 5 degrees in angulation and 2.3 mm in linear distance between the implant placement and the preoperative plan [14]. For inexperienced clinicians, inadequate training in guided surgery may result in even greater deviations and, in severe cases, complications such as nerve injury. Common issues include suboptimal virtual implant pathway orientation, poor fit or fracture of surgical guides, and intraoperative changes to the treatment plan. Therefore, integrating automated and standardized methods of assessment, planning, and execution, such as the use of surgical guides, the establishment of unified diagnostic criteria, and systematic investigation of risk factors, will be critical to improving implant success rates, minimizing complications, and ensuring patients’ long-term oral health [15].

In clinical practice, DPR is commonly used as a preliminary, broad-overview screening tool and is widely adopted across both medical centers and community dental clinics [16]. Moreover, DPR can be used during the initial consultation to quickly communicate potential implant directions to patients, facilitating doctor–patient communication [17]. Compared with CBCT, which is considered the gold standard for three-dimensional evaluation and computer-assisted guided implant surgery, DPR offers advantages such as lower radiation dose, lower cost, and greater accessibility [18]. Therefore, while CBCT remains indispensable for complex cases and computer-assisted guided implant therapy [19]. DPR-based analysis holds clinical value in the early assessment stage, particularly for communication and educational purposes [20]. Recent systematic reviews have highlighted the growing evidence for AI in DPR interpretation [21,22] and implant pathway planning [23], showing improved precision and predictability over conventional methods. Advances in deep learning, particularly CNNs [24] and object detection [25], have enabled automated identification of anatomical structures and pathologies. Balel et al. [26] reported YOLOv8-based implant segmentation with >91% precision and F1-scores up to 0.966, while Wang et al. [27] demonstrated AI’s capability for standardized multinational DPR analysis across diverse populations.

However, to the best of our knowledge, few studies have specifically investigated the feasibility of AI-assisted estimation of implant pathway direction based on DPR images as an auxiliary step prior to CBCT confirmation. We hypothesize that deep learning models can effectively identify edentulous regions and provide an initial prediction of implant pathway orientation from DPR with clinically acceptable accuracy and stability. Moreover, this study aims to develop and validate a visualized AI-assisted framework for DPR-based implant pathway detection, providing a practical adjunct to pre-assessment discussion and educational explanation in routine clinical practice. In this study, we propose a visualized AI-assisted implant placement pathway detection framework for DPR. Our approach integrates the YOLO model to accurately annotate adjacent teeth in edentulous regions of DPR and predict the preliminary implant pathway. The process employs YOLO to detect missing-tooth regions, followed by image processing techniques to enhance image quality. Subsequently, the Oriented Bounding Box (OBB) method is applied to extract pixel location information about neighboring healthy teeth. Based on these data, an algorithm derives clinically suitable implant placement pathways. This study uses DPR as the dataset to present the first deep-learning-based approach for implant pathway orientation prediction on DPR.

## 2. Materials and Methods

**Figure 1:**
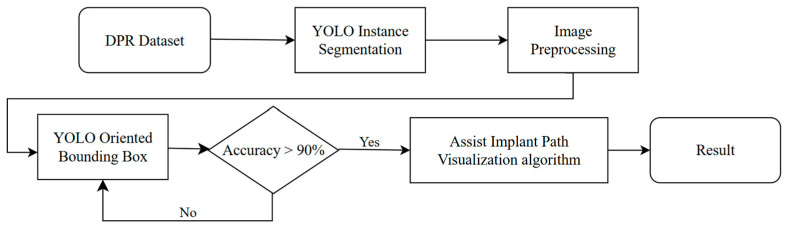
Implant pathway orientation evaluation flowchart.

This section introduces the proposed automated auxiliary technique for implant pathway orientation detection on DPR. The overall research workflow is shown in Figure 1. We used two types of YOLO models, and with the visualized implant pathway orientation evaluation technique developed in this work, these models are employed to perform the detection of missing teeth and determine the preliminary implant pathway orientation.

### 2.1. Dataset Collection and Annotation

The data in the DPR dataset was collected from five dental teams across different branches of Chang Gung Memorial Hospital in Taiwan, ensuring representation from multiple clinical centers and reducing potential bias from a single-site collection. A total of 500 DPRs were included from patients aged 20 to 65 years, with a male-to-female ratio of 53:47. Ethical approval was obtained from the Institutional Review Board of Chang Gung Memorial Hospital (IRB: 202301730B0), ensuring compliance with regulatory and ethical standards. All DPRs were acquired using standardized exposure protocols; the exposure time was incrementally adjustable from 0.03 to 3.2 s, depending on clinical needs. Digital sensors with a size of 31.3 × 44.5 mm were used, resulting in an image resolution of either 2100 × 1032, saved in DCI format, with a development time of ≤5 s. To minimize variability in image geometry, an X-ray indicator ring and a sensor holder were applied for all subjects to standardize the angle between the X-ray cone and the sensor.

**Figure 2:**
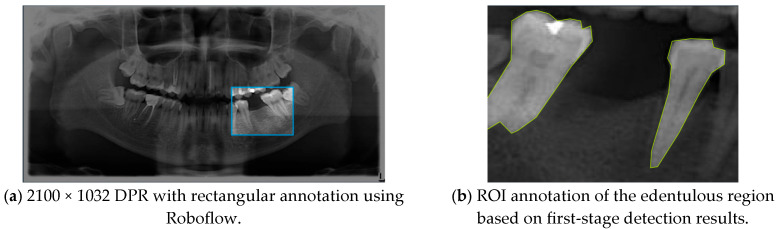
Visualization of the two-stage YOLO annotation method for edentulous region detection.

The database was annotated by five senior dentists, each representing one of the participating teams, all of whom had more than five years of clinical implantology experience. To ensure rigor, only DPRs with a single-tooth edentulous site and two adjacent neighboring teeth were included, while cases involving multiple missing teeth or periodontal bone loss in both adjacent teeth (with potential need for extraction) were excluded. Each senior dentist performed the annotations independently, without influence from others, and the final ground truth was determined through majority voting to guarantee consistency and reliability. In the annotation stage, the Roboflow annotation tool labeled the edentulous regions and the two adjacent teeth with rectangular bounding boxes (Figure 2a), serving as the basis for subsequent evaluation. In the second stage, the YOLO-based cropped outputs and segmented DPR subsets were used for region of interest (ROI) annotation and training (Figure 2b), enabling the derivation of optimal implant placement pathways.

Moreover, 500 DPR databases were divided into a training set and a test set, and 50 DPRs were preserved to compare our AI-assisted framework and the dentist’s ground truth. However, the relatively limited number of original images raised concerns about potential overfitting during model training, which could compromise generalization [28]. Thus, image augmentation techniques enhanced dataset diversity and model robustness, doubling the dataset size. The augmentation methods used in this study included brightness adjustment (−25% to +25%), exposure modification (−15% to +15%), and random Gaussian noise addition (−15% to +15%), simulating variations in patient exposure conditions during DPR acquisition. The final dataset separation ratio is shown in Table 1.

### 2.2. Extraction of Missing Teeth by Deep Learning Method

DPRs are widely used in dentistry. However, uneven thickness distribution of the jawbone often leads to high noise levels, affecting overall image quality. Certain dental regions and the alveolar ridge exhibit pixel values that are too similar, making image recognition more challenging. Moreover, teeth occupy only about one-quarter to one-third of the entire DPR, meaning that many irrelevant regions do not contribute to the analysis and may even interfere with model interpretation. Since DPR includes multiple teeth, errors in tooth localization may also occur during subsequent model processing. This study introduces YOLO [29], a real-time object detection algorithm, to address these challenges. Its core concept reformulates object detection as a single regression problem, enabling simultaneous prediction of object classes and locations within a single neural network inference. This method significantly reduces computation time and achieves real-time detection performance compared to traditional region proposal approaches.

In clinical implant pathway orientation, dentists typically determine the placement direction and position of implants based on the alignment, occlusion, and angulation of adjacent teeth [30]. Therefore, digital implant analysis requires accurate acquisition of each tooth’s inclination and spatial orientation. This study adopted the OBB technique to recognize orientation-specific dental structures. For the ROI images, the YOLO-OBB model was employed in subsequent experiments to extract the rotational matrix coordinates of the teeth and calculate the implant placement pathway. The hardware and software specifications used for training the YOLO and YOLO-OBB models are summarized in Table 2.

#### 2.2.1. YOLO Architecture

In the first stage, the YOLO architecture series detected edentulous regions. YOLO is a one-stage object detection model whose core advantage lies in integrating object localization and classification within a single neural network, significantly improving computational efficiency and maintaining accuracy. The YOLO architecture consists of three core modules: Backbone, Neck, and Head. The Backbone utilizes a deep convolutional neural network for multi-scale feature extraction, effectively capturing both local and global information from dental images, such as tooth contours and the surrounding bone structures in edentulous regions. The Neck applies mechanisms such as Feature Pyramid Networks (FPNs) or Path Aggregation Networks (PANs) to achieve cross-layer feature fusion. This enhances the model’s ability to detect dental morphology and missing-tooth characteristics at different scales, reducing missed detections caused by variations in image resolution or visual contrast. Finally, the Head module produces the ultimate bounding box and class predictions, converting the extracted features into clinically interpretable annotations of edentulous regions. Given that DPRs are characterized by high-density structural overlaps and subtle grayscale variations, accurately identifying edentulous regions under such complex conditions remains a significant challenge. By leveraging end-to-end training and efficient feature fusion, the YOLO framework improves detection feasibility for clinical decision support while preserving fine image details. Therefore, this study adopts the most recent four YOLO versions, YOLOv9, YOLOv10, YOLOv11, and YOLOv12, for comparative evaluation and experimental analysis. The architectures of these models are described as follows.

1. YOLOv9 integrates Programmable Gradient Information (PGI) and a Generalized Efficient Layer Aggregation Network to address information loss in deep networks while maintaining high detection accuracy, particularly for small objects [31].
2. YOLOv10 employs a dual assignment strategy, lightweight head, and spatial channel decoupled downsampling, reducing inference time and minimizing information loss during feature extraction [32].
3. YOLOv11 replaces traditional C2f blocks with C3k2 blocks to improve gradient flow and computational efficiency; integrates the SPPF module for multi-scale context capture; and incorporates the C2PSA attention mechanism to enhance spatial feature representation [33,34].
4. YOLOv12 adopts an attention-centric design with the A^2^ regional attention module for dynamic global–local feature capture; incorporates the R-ELAN architecture for enhanced feature aggregation and gradient stability; and integrates Flash Attention and adaptive MLP ratio optimization, achieving superior inference speed and detection accuracy over previous versions [35].

#### 2.2.2. YOLO-OBB Architecture

**Figure 3:**
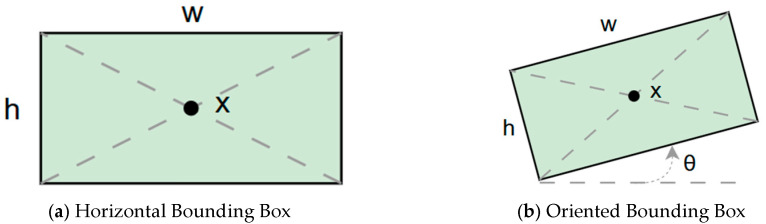
Bounding Box used in YOLO detection. (**a**) Horizontal Bounding Box (HBB); (**b**) Oriented Bounding Box (OBB). Here, x represents the center point of the bounding box, w and h denote the width and height of the box, and θ indicates the rotation angle of the oriented bounding box. The dashed lines represent the diagonal axes of each box, and the arrow denotes the direction of rotation used for the angle θ.

**Figure 4:**
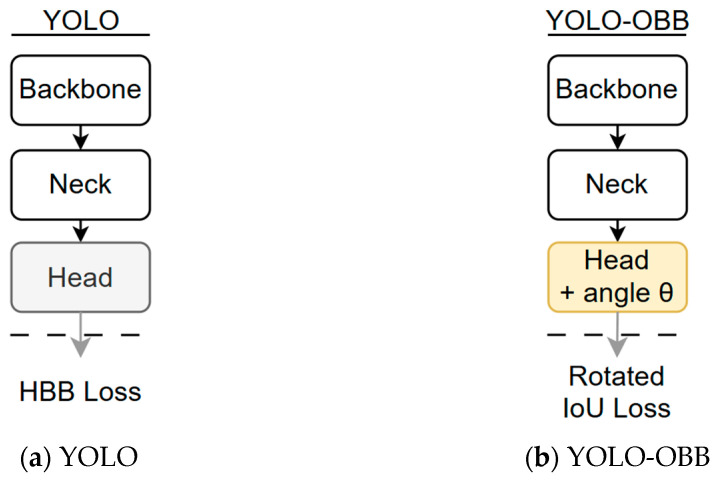
Architecture comparison between traditional YOLO and YOLO-OBB model.

In the subsequent experiments, this study adopted the OBB to recognize orientation-sensitive objects [36,37]. In practical scenarios where teeth exhibit diverse alignment directions, conventional object detection models predominantly rely on the Horizontal Bounding Box (HBB) method. However, this approach often results in low Intersection over Union (IoU) scores, thereby reducing the accuracy of localization and pose estimation. In contrast, the OBB provides a closer fit to the actual shape and angulation of teeth, thus improving the precision of detection and pose analysis. The visualized differences between the HBB and OBB are illustrated in Figure 3 and Figure 4.

The YOLO-OBB model retains the original Backbone and Neck architecture. At the same time, the Head layer is modified to predict bounding boxes and object classes and output the object’s rotation angle (θ). During training, the Rotated IoU Loss is introduced to evaluate prediction errors of OBBs, thereby enabling the model to recognize tilted or non-axis-aligned objects more accurately. This study employed YOLOv8-OBB, YOLOv10-OBB, YOLOv11-OBB, and YOLOv12-OBB as comparative models for tooth rotation matrix detection. Among these, the most robust version was selected as the primary training model, which was subsequently used to investigate further the impact of different image processing methods on model performance.

#### 2.2.3. Hyper Parameter Setting

Table 3 summarizes the hyper-parameter settings used for training the YOLO models in this study. The training was conducted for 150 epochs, with a batch size of 1 for each iteration. This configuration ensures that the model can progressively learn fine-grained dental image features even under small-batch training conditions. The optimizer adopted was AdamW, which incorporates weight decay to mitigate overfitting and improve convergence stability, particularly in angular regression tasks. The learning rate was set to 0.0005, the baseline for updating model weights. These hyper-parameter choices were designed to balance computational efficiency with model accuracy, enhancing convergence precision and generalization performance in dental clinical imaging tasks.

#### 2.2.4. Evaluation Metrices

Four primary evaluation metrics were employed to comprehensively evaluate the model’s overall performance: Accuracy, precision, recall, and mAP50/mAP50–95. These indicators reflect the model’s predictive ability and practical applicability in classification tasks from different perspectives. All evaluation metrics were calculated based on the confusion matrix [38], which consists of four core elements: true positive (TP), true negative (TN), false positive (FP), and false negative (FN). Specifically, TP represents correctly identified positive samples, TN denotes correctly identified negative samples, FP refers to negative samples misclassified as positive, and FN indicates positive samples misclassified as negative, and API,50 denotes the Average Precision. By deriving evaluation metrics from these values, we gained deeper insights into the classification accuracy of the model across different categories as well as its potential limitations. The corresponding formulas are shown in (1)–(4).

(1) $$Accuracy\;=\;\frac{TP+TN}{TP+TN+FP+FN}$$

(2) $$Precision=\frac{TP}{TP+FP}$$

(3) $$Recall=\frac{TP}{TP+FN}$$

(4) $$mAP50=\frac{1}{N}\sum_{i=1}^{N}AP_{i,50}$$

### 2.3. Tooth Image Enhancement

After the first-stage YOLO instance segmentation and prior to the second-stage YOLO-OBB processing, multiple image enhancement techniques were applied in this study to improve image quality. These enhancements were introduced to facilitate the performance of the proposed auxiliary implant pathway orientation detection algorithm.

#### 2.3.1. Bilateral Filter

**Figure 5:**
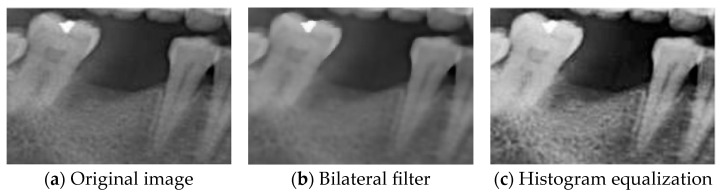
Image feature extraction.

A bilateral filter was applied to smooth the tooth regions and enhance image quality. Unlike traditional linear filters such as Gaussian filtering, which rely solely on fixed weights, the bilateral filter is a nonlinear filter that simultaneously considers both the spatial distance between pixels and the similarity of their grayscale values. Combining two Gaussian weighting functions computes a weighted average of neighboring pixel values around the target pixel. This process effectively smooths noise and artifacts while maximally preserving image edges and fine structural details, avoiding cross-edge blurring. The bilateral filtering process is defined as (5), where h(x) represents the output pixel value, f(ξ) denotes the neighboring pixel values of the input image, c(ξ,x) measures spatial proximity, s(f(ξ),f(x)) evaluates pixel intensity similarity, and k(x) is a normalization factor ensuring that the sum of weights equals one. This design reduces noise while retaining prominent edges, preventing excessive blurring that could compromise subsequent analysis [39]. As illustrated in Figure 5a,b, the bilateral filter significantly reduces image noise while preserving edge details.

(5) $$h\left(x\right)=\frac{1}{k\left(x\right)}\iint_{-\infty }^{\infty }f(\xi )c(\xi ,x)s(f(\xi ),f(x))d\xi$$

#### 2.3.2. Histogram Equalization

**Figure 6:**
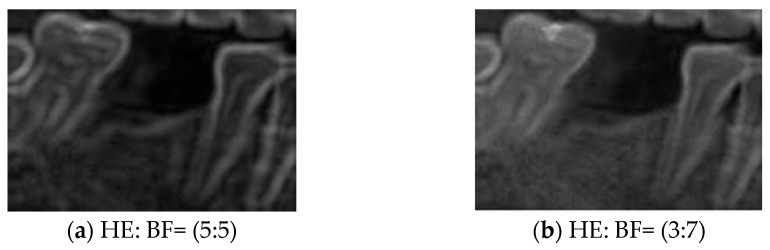
HE combines BF processing.

Histogram Equalization (HE) was applied to address the issues of uneven contrast and brightness in the original images. HE is commonly used in image enhancement cases involving low contrast or concentrated grayscale distributions. The computational procedure typically involves calculating the grayscale histogram of the original image, deriving the cumulative distribution function (CDF), and using the CDF as a mapping function to generate the enhanced image. The principle of this method is to redistribute the pixel intensity values of the image, thereby achieving a more uniform distribution of grayscale levels [40]. This approach improved overall contrast and detail visibility in this study. The transformation function of HE is defined in (6). Specifically, $s_{k}$ represents the output grayscale value, $r_{k}$ is the original pixel value, L denotes the total number of gray levels, and $p_{r}(r_{j})$ is the probability of occurrence of the j-th grayscale level. This study used two categories of images for training: BF images and enhanced images obtained through image processing. By applying HE and blending it with BF images at varying degrees, composite images (HE: BF) were generated. This approach improves the model’s adaptability to diverse clinical conditions of dental morphology, thereby enhancing its generalization performance and robustness against interference, as illustrated in Figure 6.

(6) $$s_{k}=T\left(r_{k}\right)=\left(L-1\right)\sum_{j=0}^{k}p_{r}(r_{j})$$

### 2.4. Implant Pathway Orientation Visualization Algorithm

**Figure 7:**
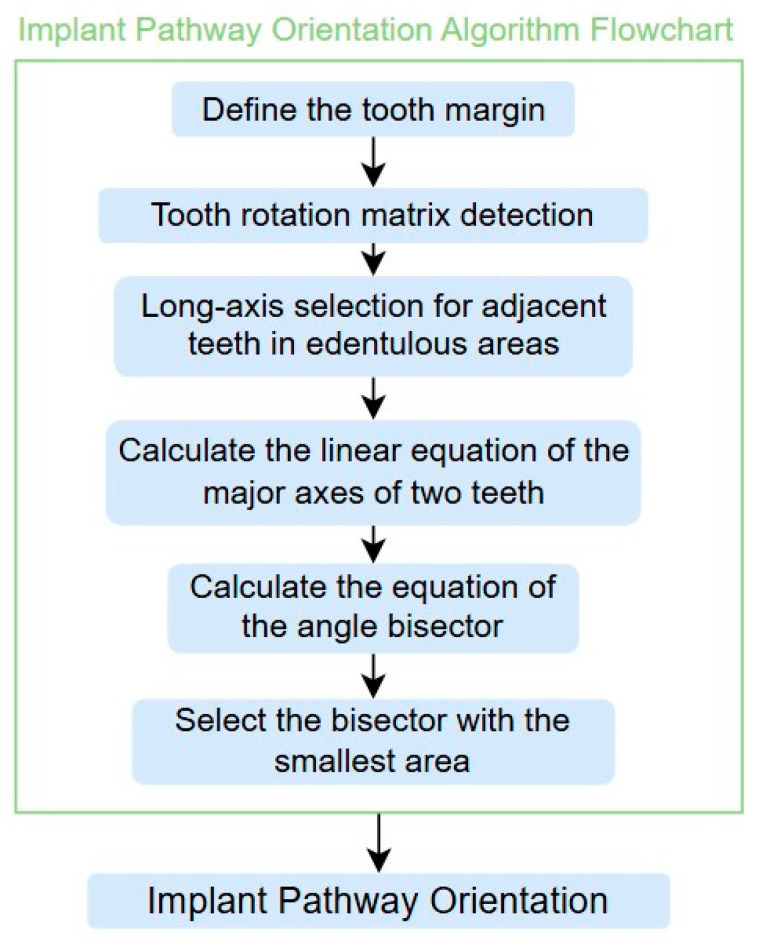
Implant Placement Pathway Algorithm Flowchart.

After obtaining the tooth contours using the YOLO-OBB algorithm, we use the algorithm developed in this study to perform implant placement pathway visualization analysis. The detailed process is shown in Figure 7. The implant placement path algorithm first defines the margins of the target teeth to establish clear analysis boundaries. It then detects the tooth rotation matrix using the YOLO-OBB algorithm to determine tooth angles. For edentulous areas, the algorithm selects the long axes of adjacent teeth as reference benchmarks. From these axes, two auxiliary segments extend along their corresponding OBB edges. These auxiliary lines are defined as shown in (7), where $A_{1}$, $B_{1}$, $C_{1}$ and $A_{2}$, $B_{2}$, $C_{2}$ are constant coefficients derived from the coordinates of two points, satisfying the general linear equation of the form Ax + By + C = 0.

(7) $$A_{1}x+B_{1}y+C_{1}=0\;A_{2}x+B_{2}y+C_{2}=0$$

(8) $$\frac{A_{1}x+B_{1}y+C_{1}}{\sqrt{{A_{1}}^{2}+{B_{1}}^{2}}}=\pm \frac{A_{2}x+B_{2}y+C_{2}}{\sqrt{{A_{2}}^{2}+{B_{2}}^{2}}}$$

**Figure 8:**
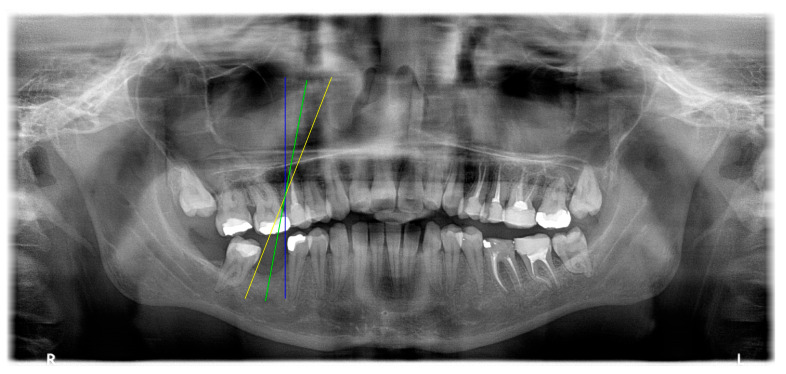
Auxiliary lines are derived from adjacent teeth, the blue and yellow line is the result of implant pathway orientation visualization algorithm, and the green is the best implant pathway orientation.

The angle bisector of the two auxiliary lines was then calculated using the angle bisector formula shown in (8), where ($A_{1}$, $B_{1}$, $C_{1}$) and ($A_{2}$, $B_{2}$, $C_{2}$) represent the coefficients of the two lines. The resulting internal angle bisector defines the preliminary implant pathway orientation derived by this framework. This bisector represents the most stable implant pathway orientation direction, ensuring that the implant avoids adjacent teeth and critical anatomical structures while maximizing bone–implant contact area. Such orientation enhances osseointegration stability and prevents complications from improper angulation, such as malocclusion or uneven stress distribution. The visualization of this process and the algorithmic design are presented in Figure 8. Finally, the auxiliary pathway generated by the algorithm was compared and overlapped with the implant pathway orientation annotated by dentists on the images to evaluate the predictive capability and accuracy of the model. The overlap results serve as a basis for further model optimization and provide valuable reference information for clinical application.

## 3. Results

This section is organized into three parts. The first subsection focuses on extracting the ROI of edentulous areas from DPR using the YOLO model to facilitate subsequent analysis. The second subsection describes the image processing methods applied to the edentulous regions and further evaluates the performance of the YOLO-OBB model adopted in this study. The third subsection validates the accuracy and feasibility of the proposed approach for implant pathway orientation by comparing the predicted results with those of dentists.

### 3.1. DPR Instance Segmentation Result

**Figure 9:**
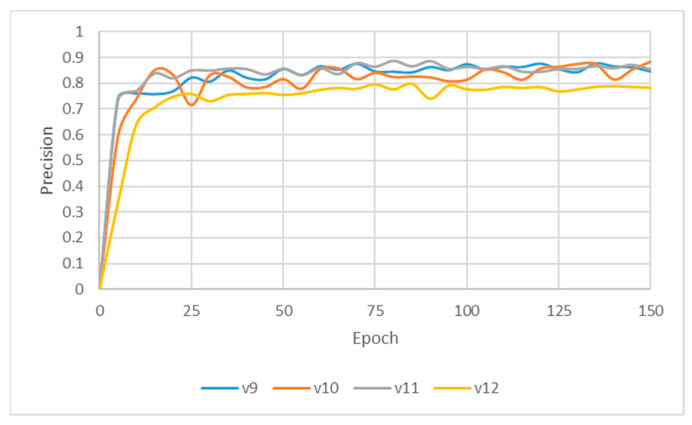
YOLO instance segmentation training processing.

This subsection evaluates the detection and segmentation results of single edentulous teeth and their two adjacent teeth on DPR images. Unlike other approaches that only extract the missing-tooth location, the proposed method incorporates the two neighboring teeth for instance segmentation, as clinical implant pathway orientation requires assessment based on adjacent dentition [13]. Four YOLO architectures, Faster R-CNN and transformer were evaluated in the experimental analysis, with the training process illustrated in Figure 9 and Table 4. Among them, the YOLOv10m model achieved the highest overall accuracy of 86.58% for single-tooth detection and segmentation, outperforming the other three YOLO models. However, its Precision and mAP50–95 were slightly lower (0.4–1%) than those of YOLOv9m. YOLOv9m demonstrated marginally superior overall performance, while YOLOv11m exhibited stronger results in Recall (88.89%) and mAP50 (89.23%), indicating its robustness in achieving more comprehensive target coverage. Considering overall performance and practical applicability, this study compared YOLOv9m and YOLOv11m in the segmentation task. YOLOv9m achieved higher Precision (88.86%) and mAP50–95 (75.34%), suggesting that it was more effective in reducing false positives and maintained stable accuracy across varying IoU thresholds. Moreover, we conducted a statistical analysis to ensure the reliability of the model’s performance. We used *p*-values, McNemar’s test, and a paired t-test to evaluate different models. The results indicate that YOLOv9m and YOLOv11m achieved comparable performance without significant differences. At the same time, Faster R-CNN and Swin-transformers showed significantly lower performance, confirming the robustness of the proposed YOLO-based framework. In terms of training efficiency, YOLO models required shorter training times per epoch compared to Faster R-CNN and Swin-transformer. Among the YOLO variants, YOLOv11m achieved a balanced training time of 11:39 ms, while YOLOv9m and YOLOv12m required 14:47 ms and 12:58 ms, respectively. In contrast, Faster R-CNN (18:15 ms) and Swin-transformer (22:08 ms) showed substantially longer training times, indicating higher computational demands.

We conducted 10-fold cross-validation to validate the performance of the YOLOv9m model under different data splits. Repeated stratified holdout [41] was employed during each fold’s evaluation to mitigate bias caused by variations in data distribution. As shown in Table 5, the metrics across the ten test sets exhibited consistent performance, demonstrating the model’s robust stability. YOLOv9m achieved average performance metrics of: Accuracy 85.38%, Precision 88.84%, Specificity 86.76%, Sensitivity 85.72%. The 95% confidence intervals (95% CIs) for these metrics are narrow, with minimal variation in mean ± standard deviation (mean ± SD). This indicates limited performance variation across different dilation factors, reflecting high robustness and consistency. Further statistical tests reveal that most metrics have *p*-values significantly <0.02, confirming that performance differences in YOLOv9m are statistically significant rather than random fluctuations.

Moreover, we further employed one-way analysis of variance (ANOVA) and Tukey’s post hoc test to determine whether there were performance differences between the best YOLO model faster R-CNN and swin-transformer. The results are shown in Table 6 and Table 7, which show the mean accuracy (MEAN), sample size (n), within-group sum of squares (ss), degrees of freedom (df), mean squares (MS), F-ratio (F) and Tukey HSD critical value (q-crit). Table 7 summarizes the results of a one-way analysis of variance (ANOVA) conducted to compare the performance among the three YOLO models. The analysis revealed a significant difference between groups (F = 18.37, *p* < 0.001). Specifically, the between-group variance (SS = 0.0485, df = 2, MS = 0.0242) was considerably larger than the within-group variance (SS = 0.0118, df = 27, MS = 0.00044), resulting in a high F-value. This indicates that at least one model performed significantly differently compared to the others. The total variance explained by the analysis was 0.0603 (df = 29).

Table 7 shows Tukey’s post hoc test for the three models (YOLOv9m, Faster R-CNN, Swin-Transformer) under 10-fold cross-validation. The results show that YOLOv9m achieves the highest mean accuracy (0.8538), Faster R-CNN ranks in the middle (0.7591), and Swin-Transformer has the lowest accuracy (0.7039). Each group contained 10 samples, corresponding to 9 degrees of freedom; the total sum of squares was 0.02050 with 27 degrees of freedom. At the significance level α = 0.05, the Tukey HSD critical value was 3.50, which served as the benchmark for subsequent pairwise comparisons. The post hoc pairwise comparison results indicate that the difference in average accuracy between YOLOv9m and Faster R-CNN is 0.0947, with a test statistic q = 7.17. This value exceeds 3.50, confirming a statistically significant difference. Similarly, the difference between YOLOv9m and Swin-Transformer is even more pronounced (mean difference 0.1499, q = 10.71), also reaching statistical significance. Furthermore, the difference between Faster R-CNN and Swin-Transformer is 0.0552, corresponding to q = 4.42, which also exceeds the critical value, indicating a significant difference between them.

**Figure 10:**
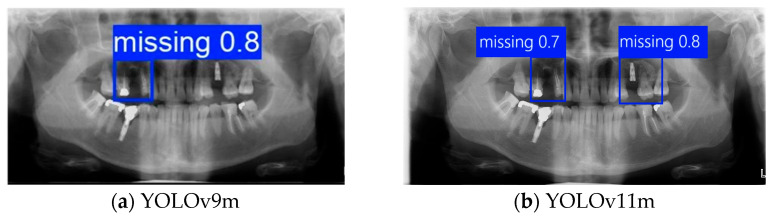
Instance segmentation result comparison.

Further analysis revealed that although YOLOv11m performed slightly better on specific metrics, it was more prone to detection box shifts or overlaps in test images with complex backgrounds or blurred tooth boundaries, leading to reduced segmentation accuracy. As shown in Figure 10b, YOLOv11m produced detections with higher confidence scores; however, the predicted locations often deviated from the actual tooth positions. In contrast, Figure 10a illustrates that YOLOv9m, despite yielding slightly lower confidence scores, achieved superior localization accuracy with fewer false positives. This advantage was particularly evident in challenging cases involving blurred edges or narrow interdental spacing, where YOLOv9m consistently delivered stable predictions. The detection boxes generated by YOLOv9m provided higher accuracy and adhered more closely to the actual tooth contours, demonstrating greater stability and practical applicability. These strengths make YOLOv9m more suitable for subsequent fine-grained image segmentation and detailed dental structure analysis. Therefore, YOLOv9m was ultimately selected as the primary model for the following experiments, and all further applications and performance evaluations were conducted based on its outputs.

### 3.2. YOLO-OBB Segmentation Result

**Figure 11:**
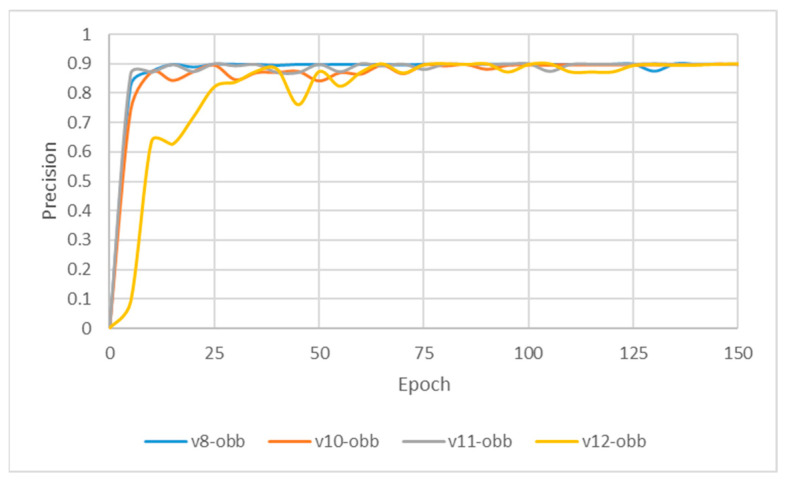
YOLO-OBB-based model training processing.

This subsection evaluates the effectiveness of image enhancement techniques applied to the YOLO-OBB model for detecting the two adjacent teeth. In this study, a composite image processing strategy (HE: BF = 3:7) was integrated to assess the training performance of the YOLO-OBB framework, with the workflow illustrated in Figure 11 and the results summarized in Table 8. The Accuracy of YOLOv8n-OBB, YOLOv10n-OBB, YOLOv11n-OBB, and YOLOv12n-OBB remained consistent within the range of 89.5–89.9%. Precision and Recall were also close to 90%, indicating stable performance regarding correct prediction and comprehensive coverage. For the mAP50 metric, all models achieved a value of 89.50%, reflecting highly consistent detection capability across versions. However, more pronounced differences emerged in the mAP50–95 evaluation. YOLOv8n-OBB and YOLOv11n-OBB achieved higher scores of 78.75% and 77.48%, respectively, outperforming YOLOv10n-OBB (71.73%) and YOLOv12n-OBB (70.14%). Although the four versions demonstrated comparable Accuracy, Precision, and Recall, the variation in mAP50–95 highlights performance discrepancies under stricter IoU thresholds, suggesting that further optimization is needed to enhance robustness in challenging detection scenarios. Moreover, the training time per epoch varied across the YOLO-OBB models, ranging from 21:03 ms (YOLOv8n-obb) to 26:54 ms (YOLOv12n-obb). YOLOv8n-obb and YOLOv10n-obb demonstrated the shortest training times, while YOLOv12n-obb required the longest.

**Figure 12:**
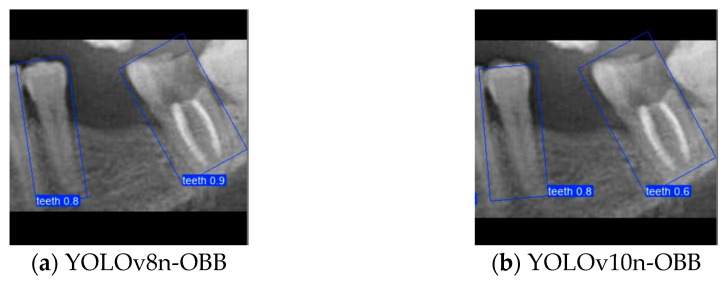
YOLO-OBB segmentation comparison.

Although YOLOv10n-OBB demonstrated a slight advantage in overall classification accuracy, YOLOv8n-OBB achieved a relatively higher mAP50–95 of 78.75%. Furthermore, as illustrated in Figure 12, YOLOv8n-OBB generated predictions with higher confidence scores, and its detection boxes more comprehensively encompassed critical surrounding structures of the teeth, including both the crown and root regions. In contrast, YOLOv10n-OBB, despite exhibiting better classification accuracy, often produced detection boxes with insufficient coverage or positional deviations, which could compromise the accuracy and stability of subsequent localization and contour segmentation tasks. Considering both model performance and practical image application, the stable and reliable predictions provided by YOLOv8n-OBB better meet the requirements of fine-grained dental feature recognition. Moreover, its robustness is advantageous for downstream applications such as auxiliary line generation and implant pathway orientation.

Table 9 presents the detection performance of the YOLOv8-OBB model under different image processing techniques. The original images (O) showed relatively lower performance, with an Accuracy of 81.80% and mAP50–95 of only 70.75%, indicating limited robustness of unprocessed data across multiple IoU thresholds. Applying BF resulted in modest improvements across all metrics, particularly Precision (84.79%) and Recall (83.98%), demonstrating the effectiveness of noise reduction and edge preservation for dental image detection. Further enhancement using HE increased Accuracy to 84.77% and mAP50–95 to 74.60%, suggesting that improved contrast and brightness distribution benefited feature extraction of tooth boundaries. When HE and BF were combined at a 5:5 ratio, performance improved further, achieving an Accuracy of 85.78%, a Recall of 85.98%, and mAP50–95 of 75.42%. Notably, the HE: BF = 3:7 composite images yielded the best overall results, with Accuracy reaching 89.80%, Precision and Recall near 90%, and mAP50–95 improving to 78.75%. This highlights the complementary strengths of HE and BF in enhancing model generalization and stability.

### 3.3. Comparison with Clinical Ground Truth and AI-Assisted Framework

This subsection presents a comparison between the dentist-defined clinical gold standard and the predictions generated by our AI-assisted framework to evaluate the reliability of implant pathway orientation. The result is shown in Table 10, the preserved DPR validation set was used for verification, and the AI-assisted framework results were overlaid onto the original DPR images to provide a clear visualization. The AI-assisted framework pathways (green lines) were highly consistent with the dentists’ ground-truth annotations (black lines). Quantitatively, the mean squared error (MSE) between the predicted implant direction and the dentists’ planned results was only 1.537° across multiple test images. This minimal deviation validates the proposed system’s technical feasibility. It highlights its clinical potential to provide accurate and stable guidance for implant placement, thereby reducing the risk of misalignment and supporting efficient preoperative orientation.

## 4. Discussion

Ours is the first study to propose an AI-assisted framework for preliminary implant pathway evaluation in DPR. Our framework is not intended to replace CBCT or standard clinical planning procedures. Instead, it serves as a preliminary proof-of-concept study demonstrating the feasibility of AI-assisted estimation of implant pathway orientation on two-dimensional DPR. This approach may be valuable for training, educational visualization, and early-stage communication between clinicians and patients, offering an auxiliary tool to support rather than substitute comprehensive CBCT-based planning.

Previous studies have explored a variety of AI methods beyond YOLO architecture, but each carries specific limitations. U-Net segmentation models have shown high accuracy on CBCT, yet their dependence on costly imaging with higher radiation exposure restricts routine use [42]. R-CNN-based approaches, including Faster R-CNN and Mask R-CNN, achieve reliable detection on DPR but are computationally demanding and less efficient for real-time applications [43]. These limitations highlight the need for approaches like YOLO-OBB that balance efficiency, accuracy, and clinical applicability in DPR. Compared with the previous study, our framework employs YOLO models to detect edentulous regions, followed by image preprocessing, and integrates pixel location information of neighboring healthy teeth to derive clinically appropriate implant pathway recommendations. The proposed system can provide preliminary implant suggestions within a short time during a patient’s initial consultation, assisting dentists in making rapid treatment plans, reducing diagnostic workload, and improving clinical efficiency. This study uses DPR as the dataset to introduce an innovative deep learning-assisted diagnostic system. The three main innovations and contributions to this work are as follows:

This study is the first work to introduce a deep learning-based framework for preliminary implant pathway orientation on DPR, offering support for both novice and experienced dentists.

We adopted the YOLO-OBB model to address the limitation of conventional horizontal bounding boxes, enhancing the detection of tilted tooth structures and achieving robust, high-precision performance.

We developed a novel implant pathway visualization algorithm, which achieved an average angular deviation of only 1.537° compared with dentists’ clinical planning, confirming the feasibility of our system to effectively support pathway decision-making in real clinical contexts.

This study demonstrates the feasibility of using YOLO-OBB for implant pathway orientation on DPR; however, it does not guarantee extremely low error levels in all situations. Implant pathway orientation errors remain a common challenge in clinical practice. In our validation using the test set, approximately 2% of DPRs showed insufficient image contrast, resulting in blurred tooth margins. This led to localization errors by the deep learning model and subsequent pathway miscalculations, with the maximum angular deviation reaching 3.42°. Such challenges are not unique to our study; for instance, Kaewsiri et al. [44] reported angular deviations of up to 4.55° using computer vision–assisted implant pathway orientation. From a clinical perspective, angular deviations within about 3° are generally considered acceptable and unlikely to compromise implant stability or surgical outcomes [45], whereas deviations greater than 5° may increase the risk of cortical bone fenestration or damage to adjacent anatomical structures [3]. Although the error levels in our study remain within a clinically tolerable range, future work will focus on improving DPR normalization and preprocessing techniques to further reduce failure cases and enhance robustness.

Moreover, the present study was limited to single-tooth edentulous cases, which means the model is currently unable to evaluate implant pathways involving multiple missing teeth or more complex clinical scenarios. This restriction narrows the clinical applicability and should be addressed in future work. Additionally, since DPR provides only two-dimensional information without volumetric bone data, the proposed framework should be considered as an early-phase exploratory study focusing on technical feasibility rather than a clinically deployable system. Future work will integrate CBCT and multimodal image fusion to address these limitations, alongside refined preprocessing techniques and explainable AI (XAI) to improve transparency and reliability. Expanding the dataset with greater diversity in bone quality, pathology, and implant systems will further enhance model generalization. Moreover, we provided our codes in GitHub for public access, enabling other researchers to reproduce our experiments and validate the proposed framework (https://github.com/030didi/Dental-implant-detection, accessed on 10 January 2025). These strategies are expected to improve predictive accuracy and provide dentists with a more reliable tool for implant orientation, reducing clinical risks and advancing intelligent dental diagnostic systems.

## 5. Conclusions

This study focused on preliminary implant pathway orientation using DPR, given its accessibility, lower radiation dose, and cost-effectiveness. This is the first work to propose a deep learning-based approach for implant pathway orientation, incorporating OBB annotation to enhance the recognition of tilted tooth structures, which may provide clinicians with supportive information to improve precision and efficiency during implant procedures. The experimental results demonstrated that YOLOv9m and YOLOv8n-obb achieved balanced performance in recognition capability and accuracy. Training with unprocessed raw images yielded superior accuracy, suggesting advantages for model learning and prediction. Overall, the models showed high accuracy and stability in detecting edentulous regions and predicting implant pathways, confirming the method’s feasibility for clinical applications. This work provides a promising auxiliary tool for dental diagnostics and establishes a foundation for the future development of computer-assisted implant pathway orientation and intelligent dental treatment systems.

Nevertheless, this study is limited to DPR-based preliminary orientation and single-tooth edentulous cases. Future work will focus on expanding the dataset, integrating CBCT and multimodal imaging, and exploring explainable AI to further enhance clinical reliability and applicability.

## Figures and Tables

**Table 1:** Image number comparison before and after dataset augmentation.

Dataset Augmentation	Training Set (70%)	Test Set (30%)	Validation Set
Before	315	135	50
After	630	270	100

**Table 2:** The hardware and software platform version.

**Hardware Platform**	**Version**
CPU	AMD Ryzen™ R7-7700@3.80 GHz
GPU	NVIDIA GeForce RTX 3070 8G
DRAM	64 GB
**Software Platform**	**Version**
Python	3.9.31
PyTorch	2.4 + cu121
CUDA	12.1

**Table 3:** Hyper-parameter setting.

Hyper-Parameter	Value
Epochs	150
Batch size	1
Learning rate	0.0005
optimizer	AdamW

**Table 4:** YOLO model training result for DPR instance segmentation.

	Accuracy	Precision	Recall	mAP50	mAP50–95	Training Time (m:s)	*p*-Value	McNemar’s Test	Paired *t*-Test
YOLOv9m	85.60%	88.86%	86.67%	88.45%	75.34%	14:47	0.045	-	-
YOLOv10m	80.20%	83.09%	86.67%	88.59%	75.62%	13:32	0.082	0.091	0.088
YOLOv11m	86.58%	87.64%	88.89%	89.23%	74.94%	11:39	0.046	0.052	0.048
YOLOv12m	78.88%	83.79%	84.44%	85.16%	60.37%	12:58	0.117	0.103	0.110
Faster R-CNN	75.98%	79.63%	80.01%	78.23%	59.65%	18:15	0.189	0.2325	0.4295
Swin-transformer	70.98%	71.47%	73.86%	67.89%	54.62%	22:08	0.207	0.4211	0.4238

**Table 5:** 10-fold cross validation with YOLOv9m.

Test Set	Accuracy	Precision	Specificity	Sensitivity	IoU
1	0.8578	0.8978	0.8695	0.8645	0.8402
2	0.8485	0.8985	0.8694	0.8560	0.8225
3	0.8513	0.8813	0.8524	0.8559	0.8395
4	0.8693	0.8693	0.8695	0.8412	0.8221
5	0.8464	0.8964	0.8695	0.8609	0.8344
6	0.8481	0.8981	0.8695	0.8675	0.8459
7	0.8516	0.8816	0.8695	0.8572	0.8419
8	0.8485	0.8985	0.8674	0.8687	0.8480
9	0.8476	0.8976	0.8695	0.8590	0.8294
10	0.8690	0.8640	0.8695	0.8406	0.8262
Average	0.8538	0.8884	0.8676	0.8572	0.8350
mean ± SD	0.8538 ± 0.0082	0.8884 ± 0.0126	0.8676 ± 0.0051	0.8572 ± 0.0092	0.8350 ± 0.0090
*p*-value	0.0122	0.0098	0.0101	0.0113	0.0128
95% CI	[0.8487,0.8589]	[0.8806,0.8962]	[0.8644,0.8708]	[0.8515,0.8629]	[0.8294,0.8406]

**Table 6:** Three models of One-Way Analysis of Variance (ANOVA).

Source	SS	df	MS	F	*p*-Value
Between Groups	0.0485	2	0.0242	18.37	<0.001
Within Groups	0.0118	27	0.00044	–	–
Total	0.0603	29	–	–	–

**Table 7:** Tukey honestly significant difference test.

Groups	MEAN	n	SS	df	q-Crit
YOLOv9m	0.8538	10	0.00742	9	–
Faster R-CNN	0.7591	10	0.00683	9	–
Swin-Transformer	0.7039	10	0.00625	9	–
Total	–	30	0.02050	27	3.50

**Table 8:** YOLO-OBB training result.

	Accuracy	Precision	Recall	mAP50	mAP50–95	Training Time (m:s)
YOLOv8n-obb	89.80%	89.82%	89.98%	89.50%	78.75%	21:03
YOLOv10n-obb	89.85%	89.87%	89.98%	89.50%	71.73%	21:14
YOLOv11n-obb	89.79%	89.98%	89.81%	89.50%	77.48%	25:12
YOLOv12n-obb	89.52%	89.54%	89.98%	89.50%	70.14%	26:54

**Table 9:** Comparison with different image processing based on YOLOv8-OBB model.

	Accuracy	Precision	Recall	mAP50	mAP50–95
O	81.80%	81.82%	81.98%	81.50%	70.75%
BF	83.77%	84.79%	83.98%	82.50%	72.60%
HE	84.77%	85.79%	84.98%	84.50%	74.60%
HE: BF (5:5)	85.78%	85.80%	85.98%	85.50%	75.42%
HE: BF (3:7)	89.80%	89.82%	89.98%	89.50%	78.75%

**Table 10:** Comparison between model-predicted pathways and dentist-planned pathways.

YOLO-OBB result						
Validation Image 1–6	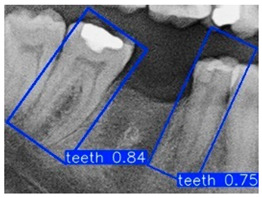	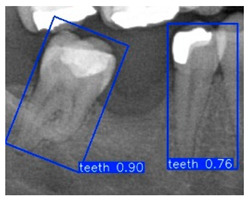	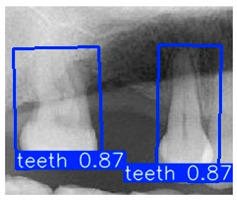	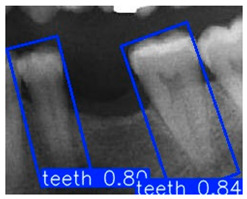	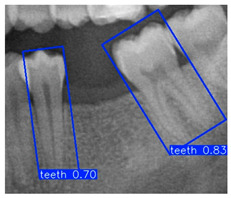	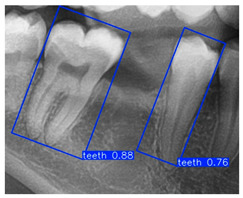
Accuracy	84.13% 75.18%	90.29% 76.49%	87.38% 87.27%	80.40% 84.48%	70.11% 83.41%	88.46% 76.33%
AI-assisted and implant path visualization result						
Validation Image 1–6	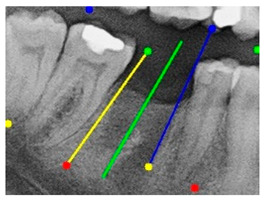	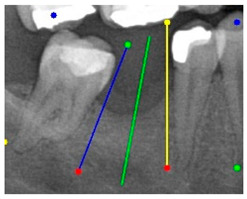	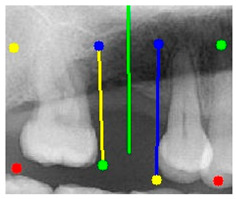	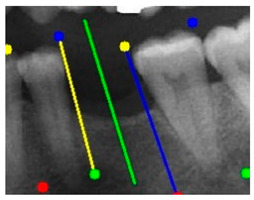	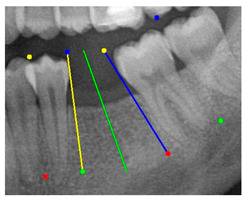	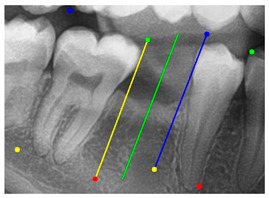
Comparison with dentist’s ground truth (black line) and our framework (green line)						
Validation Image 1–6	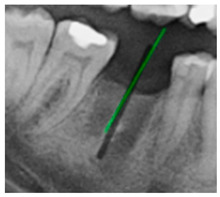	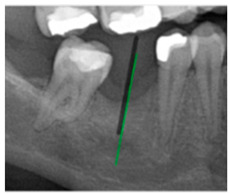	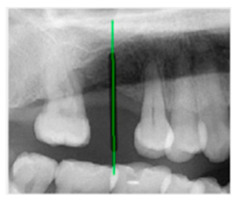	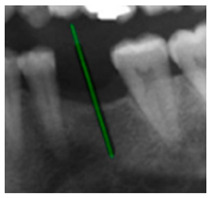	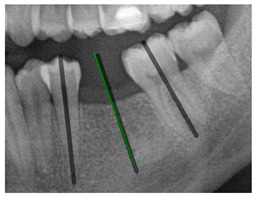	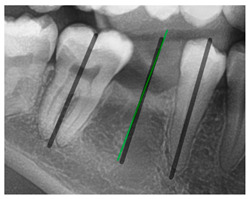
MSE	3.59°	1.29°	1.01°	0.41°	0.80°	2.12°

Colored annotations indicate different visualization purposes: (1) The **yellow** and **blue lines** represent auxiliary reference lines. (2) The **green lines** represent the implant pathway (predicted by the model). (3) All **colored dots** indicate auxiliary points used for alignment and measurement.

## Data Availability

The dataset can be made available from the corresponding author upon reasonable request only for research purposes, subject to approval by the Institutional Review Board.

## References

[1] French D., Ofec R., Levin L. (2021). Long term clinical performance of 10,871 dental implants with up to 22 years of follow-up: A cohort study in 4247 patients. Clin. Implant. Dent. Relat. Res..

[2] Kupka J.R., König J., Al-Nawas B., Sagheb K., Schiegnitz E. (2024). How far can we go? A 20-year meta-analysis of dental implant survival rates. Clin. Oral Investig..

[3] Takács A., Hardi E., Cavalcante B.G.N., Szabó B., Kispélyi B., Joób-Fancsaly Á., Mikulás K., Varga G., Hegyi P., Kivovics M. (2023). Advancing accuracy in guided implant placement: A comprehensive meta-analysis: Meta-Analysis evaluation of the accuracy of available implant placement Methods. J. Dent..

[4] Misch K., Wang H.-L. (2008). Implant Surgery Complications: Etiology and Treatment. Implant. Dent..

[5] Chen S.T., Buser D., Sculean A., Belser U.C. (2023). Complications and treatment errors in implant positioning in the aesthetic zone: Diagnosis and possible solutions. Periodontol. 2000.

[6] Sahrmann P., Kühl S., Dagassan-Berndt D., Bornstein M.M., Zitzmann N.U. (2024). Radiographic assessment of the peri-implant site. Periodontol. 2000.

[7] Lo Giudice R., Nicita F., Puleio F., Alibrandi A., Cervino G., Lizio A.S., Pantaleo G. (2018). Accuracy of Periapical Radiography and CBCT in Endodontic Evaluation. Int. J. Dent..

[8] Walker C., Thomson D., McKenna G. (2009). Case study: Limitations of panoramic radiography in the anterior mandible. Dent. Update.

[9] Kaasalainen T., Ekholm M., Siiskonen T., Kortesniemi M. (2021). Dental cone beam CT: An updated review. Phys. Medica.

[10] Kühl S., Payer M., Zitzmann N.U., Lambrecht J.T., Filippi A. (2015). Technical accuracy of printed surgical templates for guided implant surgery with the coDiagnostiX^TM^ software. Clin. Implant. Dent. Relat. Res..

[11] Li W.T., Li P., Piao M.Z., Zhang F., Di J. (2020). Study on bone volume harvested from the implant sites with different methods. Beijing Da Xue Xue Bao Yi Xue Ban.

[12] Do T.A., Le H.S., Shen Y.-W., Huang H.-L., Fuh L.-J. (2020). Risk Factors related to Late Failure of Dental Implant—A Systematic Review of Recent Studies. Int. J. Environ. Res. Public Health.

[13] Unsal G.-S., Turkyilmaz I., Lakhia S. (2020). Advantages and limitations of implant surgery with CAD/CAM surgical guides: A literature review. J. Clin. Exp. Dent..

[14] Dutta S.R., Passi D., Singh P., Atri M., Mohan S., Sharma A. (2020). Risks and complications associated with dental implant failure: Critical update. Natl. J. Maxillofac. Surg..

[15] Derks J., Tomasi C. (2015). Peri-implant health and disease. A systematic review of current epidemiology. J. Clin. Periodontol..

[16] Rushton V.E., Horner K., Worthington H.V. (1999). Factors influencing the selection of panoramic radiography in general dental practice. J. Dent..

[17] Özalp Ö., Tezerişener H.A., Kocabalkan B., Büyükkaplan U.Ş., Özarslan M.M., Kaya G.Ş., Altay M.A., Sindel A. (2018). Comparing the precision of panoramic radiography and cone-beam computed tomography in avoiding anatomical structures critical to dental implant surgery: A retrospective study. Imaging Sci. Dent..

[18] Fukunaga T., Kuroda S., Kurosaka H., Takano-Yamamoto T. (2006). Skeletal anchorage for orthodontic correction of maxillary protrusion with adult periodontitis. Angle Orthod..

[19] Jacobs R., Salmon B., Codari M., Hassan B., Bornstein M.M. (2018). Cone beam computed tomography in implant dentistry: Recommendations for clinical use. BMC Oral Health.

[20] Kim Y.-K., Park J.-Y., Kim S.-G., Kim J.-S., Kim J.-D. (2011). Magnification rate of digital panoramic radiographs and its effectiveness for pre-operative assessment of dental implants. Dentomaxillofacial Radiol..

[21] Thiebot N., Hamdani A., Blanchet F., Dame M., Tawfik S., Mbapou E., Kaddouh A.A., Alantar A. (2022). Implant failure rate and the prevalence of associated risk factors: A 6-year retrospective observational survey. J. Oral Med. Oral Surg..

[22] Pedersen S., Jain S., Chavez M., Ladehoff V., de Freitas B.N., Pauwels R. (2025). Pano-GAN: A Deep Generative Model for Panoramic Dental Radiographs. J. Imaging.

[23] Turosz N., Chęcińska K., Chęciński M., Sielski M., Sikora M. (2024). Evaluation of Dental Panoramic Radiographs by Artificial Intelligence Compared to Human Reference: A Diagnostic Accuracy Study. J. Clin. Med..

[24] Macrì M., D’albis V., D’albis G., Forte M., Capodiferro S., Favia G., Alrashadah A.O., García V.D.-F., Festa F. (2024). The Role and Applications of Artificial Intelligence in Dental Implant Planning: A Systematic Review. Bioengineering.

[25] Padilla R., Netto S.L., da Silva E.A.B. A Survey on Performance Metrics for Object-Detection Algorithms. Proceedings of the 2020 International Conference on Systems, Signals and Image Processing (IWSSIP).

[26] Li Z., Liu F., Yang W., Peng S., Zhou J. (2022). A Survey of Convolutional Neural Networks: Analysis, Applications, and Prospects. IEEE Trans. Neural Netw. Learn. Syst..

[27] Balel Y., Sağtaş K., Teke F., Kurt M.A. (2025). Artificial Intelligence-Based Detection and Numbering of Dental Implants on Panoramic Radiographs. Clin. Implant. Dent. Relat. Res..

[28] Goceri E. (2023). Medical image data augmentation: Techniques, comparisons and interpretations. Artif. Intell. Rev..

[29] Terven J., Córdova-Esparza D.-M., Romero-González J.-A. (2023). A Comprehensive Review of YOLO Architectures in Computer Vision: From YOLOv1 to YOLOv8 and YOLO-NAS. Mach. Learn. Knowl. Extr..

[30] Efstathiou A., Machtei E.E., Zigdon-Giladi H., Gutmacher Z., Horwitz J. (2021). The effect of a surgeon’s position on the axial inclination of dental implants placed freehand: A single-blind study. Quintessence Int..

[31] Wang C.-Y., Yeh I.-H., Liao H.-Y.M. (2024). YOLOv9: Learning What You Want to Learn Using Programmable Gradient Information. arXiv.

[32] Wang A., Chen H., Liu L., Chen K., Lin Z., Han J. (2024). YOLOv10: Real-Time End-to-End Object Detection. arXiv.

[33] Rao S.N. YOLOv11 Explained: Next-Level Object Detection with Enhanced Speed and Accuracy. Medium. https://medium.com/@nikhil-rao-20/yolov11-explained-next-level-object-detection-with-enhanced-speed-and-accuracy-2dbe2d376f71.

[34] Khanam R., Hussain M. (2024). YOLOv11: An Overview of the Key Architectural Enhancements. arXiv.

[35] Tian Y., Ye Q., Doermann D. (2025). YOLOv12: Attention-Centric Real-Time Object Detectors. arXiv.

[36] Zand M., Etemad A., Greenspan M. (2022). Oriented Bounding Boxes for Small and Freely Rotated Objects. IEEE Trans. Geosci. Remote Sens..

[37] Li S., Zhang Z., Li B., Li C. (2018). Multiscale Rotated Bounding Box-Based Deep Learning Method for Detecting Ship Targets in Remote Sensing Images. Sensors.

[38] Dobbin K.K., Simon R.M. (2011). Optimally splitting cases for training and testing high dimensional classifiers. BMC Med. Genom..

[39] Tomasi C., Manduchi R. (1998). Bilateral filtering for gray and color images. Proceedings of the Sixth International Conference on Computer Vision (IEEE Cat. No.98CH36271).

[40] Patel O., Maravi Y.P.S., Sharma S. (2013). A Comparative Study of Histogram Equalization Based Image Enhancement Techniques for Brightness Preservation and Contrast Enhancement. SIPIJ.

[41] Tanner E.M., Bornehag C.-G., Gennings C. (2019). Repeated holdout validation for weighted quantile sum regression. MethodsX.

[42] Șalgău C.A., Morar A., Zgarta A.D., Ancuța D.-L., Rădulescu A., Mitrea I.L., Tănase A.O. (2024). Applications of Machine Learning in Periodontology and Implantology: A Comprehensive Review. Ann. Biomed. Eng..

[43] Bonfanti-Gris M., Herrera A., Rodríguez-Manzaneque M.P.S., Martínez-Rus F., Pradíes G. (2025). Deep learning for tooth detection and segmentation in panoramic radiographs: A systematic review and meta-analysis. BMC Oral Health.

[44] Kaewsiri D. (2018). Comparison of the Implant Deviation Between Implants Placed Using Static and Dynamic Computer Assisted Surgery Methods. Ph.D. Thesis.

[45] Ribas B.R., Nascimento E.H.L., Freitas D.Q., Pontual A.d.A., Pontual M.L.d.A., Perez D.E.C., Ramos-Perez F.M.M. (2020). Positioning errors of dental implants and their associations with adjacent structures and anatomical variations: A CBCT-based study. Imaging Sci. Dent..

